# The relationship between sense of coherence and self-efficacy with well-being and mental health—the situation of students at a typical German university during the COVID-19 pandemic and 1 year after the lifting of social restrictions

**DOI:** 10.3389/fpsyg.2024.1457992

**Published:** 2024-10-29

**Authors:** Lisa-Marie Kösler, Stephanie Bauer, Andreas Möltner, Rainer M. Holm-Hadulla

**Affiliations:** ^1^Heidelberg University, Heidelberg, Germany; ^2^Center for Psychotherapy Research, University Hospital Heidelberg, Heidelberg, Germany; ^3^Dean’s Office of the Medical Faculty of Heidelberg University, Heidelberg, Germany; ^4^Department of General Psychiatry, University Hospital Heidelberg, Heidelberg, Germany

**Keywords:** sense of coherence, self-efficacy, mental health, well-being, COVID-19, students, social isolation

## Abstract

**Introduction:**

The present study aims to evaluate the role of sense of coherence and self-efficacy in relation to mental health and well-being, with and without social restrictions due to the COVID-19 pandemic. Additionally, this study seeks to investigate the differences in sense of coherence, self-efficacy, mental health and well-being depending on the manner in which the pandemic is being handled.

**Methods:**

A total of 27,162 students at Heidelberg University were surveyed via email at two measurement points, once with and once without social restrictions. The survey assessed sense of coherence, self-efficacy, mental health and well-being. To this end, the questionnaires Sense of Coherence Scale, Patient Health Questionnaire, WHO-Well-being-Index and the General Self-Efficacy Scale were employed. A total of 2,398 individuals participated in the initial measurement, while 701 individuals participated in the subsequent measurement.

**Results:**

The lifting of social restrictions has been associated with a notable improvement in well-being and mental health, particularly in the context of depressive syndromes. Further analysis demonstrated a positive correlation between the sense of coherence and self-efficacy at both measurement points, as well as between these two constructs and mental health and well-being. Furthermore, the sense of coherence and self-efficacy were found to account for a notable proportion of the observed variability in mental health and well-being values. Self-efficacy exhibited a significantly higher mean value at the initial measurement time point compared to the subsequent time point. In contrast, no significant difference was observed in the sense of coherence between the two measurement points.

**Discussion:**

The findings presented here illustrate the significance of social interaction, sense of coherence and self-efficacy for mental health and well-being.

## Introduction

The global COVID-19 pandemic has resulted in significant alterations to the daily lives of the majority of people worldwide. In particular, the social restrictions and the associated isolation, as well as the feelings of loneliness and the resulting strain on mental health and well-being, constituted a significant disruption for many (Schäfer et al., 2020). A study by Holm-Hadulla et al. (2021) also revealed a decline in well-being among 72.2% of the participating students at Heidelberg University during the period of social restrictions. Depressive symptoms were observed in approximately half of the sample, with one-third exhibiting indications of moderate or severe depression (Holm-Hadulla et al., 2021). The majority of students also indicated that the reduction in their well-being and mental health was triggered or exacerbated by the social restrictions imposed by the pandemic (Holm-Hadulla et al., 2021). Furthermore, another study (Isralowitz et al., 2021) also identified a correlation between the experience of depression, exhaustion, loneliness, nervousness and anger and the ongoing impact of the global pandemic.

These findings highlight the necessity for further research into the impact of the COVID-19 pandemic on mental health, particularly in younger age groups. This study aims to fulfill this task by looking at new influencing variables and also highlighting the relevance of implementing preventative measures in the context of another exceptional situation. This will make a significant contribution to the existing body of research and provide new insights into potential avenues for improvement, as this study examines the role of sense of coherence, self-efficacy and social interactions in such circumstances.

In order to gain a deeper understanding of this concept, a closer examination was conducted on the variable sense of coherence. This is not a personality trait; rather, it is a fundamental life orientation, comprising the components of comprehensibility, manageability and meaningfulness (Blättner, 2007). The relationship between the sense of coherence and mental health and well-being has been repeatedly demonstrated in previous research (Langeland et al., 2007; Nielsen et al., 2008; Siglen et al., 2007; Torinomi et al., 2022).

Another variable that has been demonstrated to be relevant in the context of mental health and well-being is self-efficacy (Bandura et al., 1988; Posadzki and Glass, 2009). As posited by Bandura (1997), self-efficacy can be defined as an individual’s belief in their capacity to successfully accomplish a specific task. For example, individuals with higher self-efficacy are better able to cope with stress (Bandura et al., 1988) and exhibit more health-promoting behaviors (Posadzki and Glass, 2009). Furthermore, self-efficacy has been found to correlate negatively with depressive symptoms and positively with the sense of coherence (Weng et al., 2008).

The objective of this study is to examine and quantify the relationships between the sense of coherence, self-efficacy, mental health and well-being, particularly in a sample of German students facing the challenges of the COVID-19 pandemic. This research may offer insights that could inform the prevention and potentially even the treatment of mental disorders. Another area of focus will be the impact of social interaction opportunities on mental health and well-being.

By situating these concepts within the context of mental health in the context of the COVID-19 pandemic, the study makes a significant contribution to the existing research literature, offering insights that can help to inform a more holistic understanding of health from a variety of perspectives.

### Mental health and COVID-19

There is a substantial body of evidence indicating a notable decline in mental health during the pandemic. This degree of reduction is considerably higher than that anticipated on a yearly basis and the observed deterioration can be largely attributed to concerns about the future (Pierce et al., 2020). In particular, the incidence of major depression has increased twofold in comparison to the preceding year, while the prevalence of anxiety disorders has risen by 1.5 times compared to the level observed in the year preceding the pandemic (Chirikov et al., 2020). Young people and women have been identified as being particularly vulnerable (Pierce et al., 2020; Talevi et al., 2020).

Loneliness was a significant factor contributing to the students’ concerns. For example, Holm-Hadulla et al. (2023) found that during the period of social isolation associated with the pandemic, almost 25% of the student sample identified loneliness and isolation as their primary concerns. In the follow-up survey, conducted 9 months after the lifting of social restrictions, the prevalence of loneliness and social isolation was reported to be only 8%. Furthermore, the levels of mental health and well-being were found to be significantly higher. This indicates that a considerable number of depressive disorders and diminished well-being are associated with social limitations (Holm-Hadulla et al., 2023). The correlation between loneliness and mental health issues is also apparent in other research (Isralowitz et al., 2021; Lim et al., 2022; Tsamakis et al., 2021; Weber et al., 2022). The impact of the ongoing pandemic on mental health is multifaceted. In addition to the direct effects, the experience of guilt and worry about unemployment may also be influenced. Furthermore, the deterioration of education and a higher substance use have been observed as consequences of the pandemic (Carson et al., 2020).

### The salutogenesis model

The salutogenesis model proposed by Antonovsky (1979) seeks to elucidate the factors that contribute to an individual’s capacity to cope with stress. Geyer (1997) posits that this model attempts to explain why some individuals are better able to cope with stress than others. In everyday life, individuals are required to manage and organize the chaos that surrounds them, as well as to identify strategies and resources that will enable them to successfully navigate the changes that occur on a daily basis (Mittelmark et al., 2017).

The model does not seek to examine the underlying mechanisms of illness (pathogenesis) per se, but rather to identify the origin of health (salutogenesis). To this end, he describes a continuum between illness and health, whereby, in his view, people possess a certain degree of health as long as they are alive (Bergström et al., 2006). In summary, it can be said that the salutogenesis model is a resource-oriented approach, as it looks for factors and mental processes that help people stay healthy despite extreme stress (Richter and Hurrelmann, 2016).

### The sense of coherence

The sense of coherence enables the individual to cope with and adapt to a life characterized by chaos. It is defined as the ability to understand the entirety of a given situation and the capacity to utilize the available resources in a manner that promotes health and well-being. While major life events may initially have a detrimental impact on an individual’s health, they can ultimately serve to enhance resilience, as the person learns to cope with stressors. It can thus be posited that negative experiences impart knowledge that an individual can draw upon in other situations (Mittelmark et al., 2017).

The sense of coherence is comprised of three facets: comprehensibility, manageability and meaningfulness (Lindström and Eriksson, 2005). Individuals with a robust sense of coherence are better able to perceive events as less stressful (comprehensibility), to mobilize their resources in response to stress (manageability) and to demonstrate motivation, desire and commitment to cope with these experiences (meaningfulness; Wolff and Ratner, 1999).

### The relationship between the sense of coherence, mental health, and well-being

There is a substantial body of evidence indicating a correlation between the sense of coherence and the mental health and well-being of individuals. For example, the sense of coherence has been found to correlate with stress (Delgado, 2007; Gustavsson-Lilius et al., 2007) and depressive syndromes (Torinomi et al., 2022). Specifically, a high sense of coherence has been associated with a lower level of depressive symptoms and anxiety (Siglen et al., 2007). Furthermore, evidence suggests that enhancing a person’s sense of coherence can mitigate symptoms and enhance overall well-being in those with mental health concerns (Langeland et al., 2007). A stronger sense of coherence was associated with a more favorable perception of overall health, at least among individuals who initially exhibited a high sense of coherence (Eriksson and Lindström, 2006). However, the sense of coherence exerts influence not only on an individual’s health but also on their well-being. Suominen and Lindström (2008) demonstrated that a robust sense of coherence can enhance subjective well-being.

Additionally, the sense of coherence has been demonstrated to predict health outcomes and to exert a significant influence on the development and maintenance of health (Goodman et al., 2000). Moreover, another study demonstrated that the sense of coherence, in conjunction with resilience, could account for nearly 50% of the variance in mental health (Knowlden et al., 2012), which corroborates Antonovsky’s assertion that a high sense of coherence is associated with enhanced health (Bergström et al., 2006). Furthermore, longitudinal studies indicate that the sense of coherence can serve as a predictive factor in relation to mental health and, consequently, well-being (Abu-Shakra et al., 2006; Suresky et al., 2008).

### Self-efficacy

As posited by Bandura (1997), self-efficacy can be defined as a person’s belief in their ability to successfully complete a given task. There are three identified sources of self-efficacy, namely personal experiences of success, learning from a model and verbal encouragement from others. Of these, personal experiences have been demonstrated to be the most significant source (Heslin and Klehe, 2006).

### The relationship between self-efficacy, mental health, and well-being

Previous studies have indicated a negative correlation between self-efficacy and psychological stress (Bandura et al., 1988), as well as between self-efficacy and depressive and anxiety symptoms (Endler et al., 2001; Lenz et al., 2002). Furthermore, an individual’s self-efficacy has been identified as a protective factor in relation to mental health (Weber et al., 2022). In contrast, low self-efficacy is associated with increased anxiety and depressive symptoms (Faure and Loxton, 2003; Kashdan and Roberts, 2004; Shnek et al., 2001) and reduced subjective well-being (Barlow et al., 2002; Bandura et al., 2003).

A study by Siddiqui (2015) revealed that self-efficacy exerted an explanatory influence on well-being to the extent of 34.9% for men and 29.7% for women (Siddiqui, 2015).

## Research hypotheses

In light of the previous research, it would be beneficial to further investigate the relationship between the sense of coherence and self-efficacy with well-being and mental health in a German student sample, particularly in the context of the ongoing impact of the pandemic. This study is therefore the inaugural investigation to analyze these relationships over the course of different measurement points. As a result, it can provide information about the influence of the variables on each other in different situations and contexts. From this, recommendations can be derived for potential interventions that may mitigate or prevent the decline in these variables during such exceptional circumstances as the pandemic.

To test the proposed correlations, the demographic data (age, gender and field of study) as well as the constructs (sense of coherence, self-efficacy, mental health and well-being) were assessed at two measurement points in university student samples. Specifically, the following hypotheses were tested:

*H1*: There is a relationship between the sense of coherence, self-efficacy, mental health and well-being within each measurement point.

*H1a*: There is a positive correlation between the sense of coherence and mental health as well as between self-efficacy and mental health within each measurement point.

*H1b*: The sense of coherence or self-efficacy can explain part of the variance in mental health scores within each measurement point.

*H1c*: There is a positive correlation between the sense of coherence and well-being as well as between self-efficacy and well-being within each measurement point.

*H1d*: The sense of coherence or self-efficacy can explain part of the variance in well-being scores within each measurement time point.

*H2*: The influence of the sense of coherence or self-efficacy on the individual differs between the two measurement points, in the sense that the constructs have a stronger influence on the individual during the COVID-19 pandemic.

*H3*: There is a positive correlation between the sense of coherence and self-efficacy within a measurement time point.

*H4*: At the second measurement point, higher values can be observed in the sense of coherence, self-efficacy, mental health and well-being compared to the first measurement point.

## Methods

Following approval by the ethics committee of Heidelberg University Hospital and the data protection officer of Heidelberg University, all students at Heidelberg University (*n* = 27,162) were invited to participate in an online survey via email. In the email, the students were invited to participate in an approximately 60-min study on the subject of the COVID-19 pandemic. The study was conducted anonymously via the online platform Limesurvey. Following receipt of the email, students were permitted to participate in the study for a period of 2 weeks.

The initial survey period commenced on 26.05.2021 and concluded on 11.06.2021. This was approximately 1.5 years after the onset of social restrictions due to the COVID-19 pandemic. During this period, the majority of individuals were engaged in online learning and social gathering places such as libraries, dining halls, cafeterias and sports facilities were also closed. A total of 2,398 participants took part in the initial measurement period. During the subsequent measurement period, which spanned from 25.05.2022 to 10.06.2022, social restrictions had been lifted for approximately 9 months. The second measurement date was selected precisely 1 year later to control for seasonal effects. At the second measurement point, a total of 701 participants were included.

To obtain more reliable data through a higher level of trust, the students’ email addresses were not saved, which is why all students were asked to take part in the survey in both years. This resulted in a subgroup at the second measurement point that stated that they had already taken part in the first survey period. However, no matching of data from the two measurement points was possible due to the lack of email addresses.

### Assessment instruments

In addition to the demographic variables of age, gender and field of study, questionnaires were administered to assess the relevant constructs of sense of coherence, self-efficacy, mental health and well-being.

The German short version of the Sense of Coherence Scale (SOC-13) (Singer and Brähler, 2007) was assessed initially. The scale for the sense of coherence is based on Antonovsky’s model of salutogenesis (Antonovsky, 1979) and comprises three subscales: comprehensibility (five items), manageability (four items) and meaningfulness (four items). The items are presented on a 7-point Likert scale, and a total score is calculated at the end of the questionnaire. The overall scale demonstrated an internal consistency of Cronbach’s alpha 0.82 at both measurement points.

Furthermore, the German version of the General Self-Efficacy Scale (Schwarzer and Jerusalem, 1995) was evaluated. This assesses the extent to which an individual believes they can cope with and exert control over a challenging situation. The scale comprises a total of 10 items, with a 4-point Likert scale as the response format. Cronbach’s alpha was 0.88 at the first measurement point and 0.87 at the second measurement point, which can be considered to be a satisfactory level of reliability.

The Patient Health Questionnaire (PHQ-D) (Löwe et al., 2003) is a screening instrument designed for the assessment of a range of mental health conditions, including somatoform syndromes, depressive syndromes, anxiety symptoms and alcohol-related disorders. The depression scale, named PHQ-9, has demonstrated satisfactory internal consistency in the data presented here. At the initial measurement point, Cronbach’s alpha was 0.87, which can be considered to be of a high standard. The internal consistency of the PHQ-9 was also deemed to be adequate at the second measurement point, with a value of 0.88. The PHQ-9 comprises nine items with a 4-point Likert scale as the response format.

Moreover, the German version of the Well-being Index (WHO-5) (World Health Organization, 1998) was employed to assess well-being at the two designated measurement points. The screening instrument comprises five items and employs a 5-point Likert scale for responses. In this study, the Cronbach’s alpha coefficient was 0.88 at both measurement points, which can be considered to indicate a high level of internal consistency. As previous publications based on the same data set (Holm-Hadulla et al., 2021; Holm-Hadulla et al., 2023; Torinomi et al., 2022) employed a percentage scale of 0–100 as the WHO-5 score, the sum score of the WHO-5 was also multiplied by four in this study, resulting in the value WHO100.

### Statistical analyses

All quantitative data analysis was conducted using R version 2023.06.0 + 421, with the following packages employed: psych, tidyverse, car, diffcor, apaTables, moments, lavaan and stargazer. These were used to calculate the descriptive parameters and statistical tests.

Correlations and regressions were calculated to analyze the relationships between sense of coherence and self-efficacy on the one hand and well-being and mental health on the other. Furthermore, the study employed *t*-tests for dependent samples to ascertain the existence of any significant differences between the variables at the two measurement points. The assumption of a normal distribution of the data was checked using a Shapiro–Wilk test. Although the result did not in every case indicate the existence of a normal distribution, *t*-tests were calculated due to the large sample size on both measurement points. The difference between the regression coefficients was calculated using *z*-tests. It should be noted, however, that the two samples were not comparable with regard to gender [*t*(1,026) = −3.97, *p* < 0.001] and field of study [*t*(1,114) = −3.00, *p* = 0.003]. No significant difference in age was observed between the two samples [*t*(1,170) = −0.85, *p* = 0.40].

## Results

At the initial measurement point, the response rate was 8.83% (*n* = 2,398). At the subsequent measurement time point, the response rate was observed to be lower at 2.58% (*n* = 701) (Tables 1–4). More information about the sample can be seen in the following tables (Tables 1–4).

**Table 1:** Age, gender and field of study of the samples at both measurement times.

Variables		2021		2022	
		N	%	N	%
Age	Under 21	662	27.6	173	24.7
	21–23	941	39.2	274	39.1
	24–25	392	16.3	143	20.4
	26–27	161	6.7	45	6.4
	Older than 27	242	10.1	66	9.4
Gender	Divers/no information	40	1.7	31	4.4
	Female	1,578	65.8	416	59.3
	Male	780	32.5	254	36.2
Field of study	Medicine	372	15.5	89	12.7
	Law	242	10.1	52	7.4
	Psychology	38	1.6	11	1.6
	Economics and social sciences	267	11.1	76	10.8
	MINT subjects	729	30.4	246	35.1
	Humanities and theology	562	23.4	175	25.0
	Other	188	7.8	52	7.4

**Table 2:** Mean values and standard deviations of the questionnaires at both measurement times.

	2021		2022	
Variables	M	SD	M	SD
1. PHQ-9	11.61	6.08	10.22	6.21
2. SOC-13	53.65	12.46	53.58	12.50
3. WHO100	37.56	21.27	47.17	21.99
4. Self-efficacy	27.49	4.96	26.98	5.05
5. Comprehensibility	19.23	5.67	18.85	5.69
6. Manageability	16.48	4.91	16.71	4.95
7. Meaningfulness	17.94	4.51	17.82	4.70

M, mean; SD, standard deviation.

**Table 3:** Correlation table of the continuous variables at the first measurement point.

Variables	1	2	3	4	5	6
1. PHQ-9						
2. SOC-13	−0.69**					
	[−0.71, −0.66]					
3. WHO100	−0.76**	0.59**				
	[−0.77, −0.74]	[0.56, 0.61]				
4. Self-efficacy	−0.44**	0.58**	0.44**			
	[−0.48, −0.41]	[0.55, 0.61]	[0.40, 0.47]			
5. Comprehensibility	−0.57**	0.89**	0.48**	0.54**		
	[−0.60, −0.54]	[0.88, 0.90]	[0.44, 0.51]	[0.51, 0.57]		
6. Manageability	−0.57**	0.85**	0.45**	0.43**	0.69**	
	[−0.59, −0.53]	[0.83, 0.86]	[0.42, 0.49]	[0.39, 0.46]	[0.67, 0.71]	
7. Meaningfulness	−0.56**	0.72**	0.53**	0.46**	0.45**	0.38**
	[−0.59, −0.53]	[0.70, 0.74]	[0.49, 0.56]	[0.42, 0.49]	[0.42, 0.49]	[0.35, 0.42]

*p < 0.05, **p < 0.01.

**Table 4:** Correlation table of the continuous variables at the second measurement point.

Variables	1	2	3	4	5	6
1. PHQ-9						
2. SOC-13	−0.69**					
	[−0.73, −0.64]					
3. WHO100	−0.76**	0.56**				
	[−0.79, −0.72]	[0.50, 0.61]				
4. Self-efficacy	−0.48**	0.60**	0.46**			
	[−0.55, −0.42]	[0.54, 0.65]	[0.39, 0.52]			
5. Comprehensibility	−0.56**	0.88**	0.44**	0.54**		
	[−0.61, −0.50]	[0.86, 0.90]	[0.37, 0.50]	[0.48, 0.60]		
6. Manageability	−0.57**	0.84**	0.42**	0.45**	0.69**	
	[−0.62, −0.51]	[0.82, 0.87]	[0.35, 0.49]	[0.38, 0.51]	[0.64, 0.73]	
7. Meaningfulness	−0.56**	0.70**	0.52**	0.45**	0.41**	0.35**
	[−0.61, −0.50]	[0.66, 0.74]	[0.46, 0.58]	[0.38, 0.51]	[0.34, 0.48]	[0.28, 0.42]

*p < 0.05, **p < 0.01.

Subsequently, the impact of the sense of coherence on mental health was evaluated through the implementation of a linear regression analysis. The model was found to be statistically significant at the first measurement time point [*F*(1, 1,979) = 1,752, *p* < 0.001] and at the subsequent measurement time point [*F*(1, 558) = 500, *p* < 0.001].

Subsequently, the same analyses were conducted with self-efficacy as the predictor variable of mental health. The regression was found to be significant at the initial measurement time point [*F*(1, 1,959) = 481, *p* < 0.001] and at the second measurement time point [*F*(1, 553) = 169.6, *p* < 0.001].

Furthermore, the impact of the sense of coherence on the students’ well-being was evaluated through a linear regression analysis. A significant model was identified through an *F*-test [*F*(1, 1,979) = 1,037, *p* < 0.001] at the first measurement point. At the second survey time point, the results were similar, with a significant *F*-test [*F*(1, 558) = 255.4, *p* < 0.001].

Similarly, the same analyses were conducted with self-efficacy as the predictor of well-being. At the initial measurement point, the value of *F*(1, 1,959) was 466.8, with a *p*-value of less than 0.001. Similarly, the values at the second measurement point were *F*(1, 553) = 149.8, with a *p*-value of less than 0.001. It can thus be concluded that all sub-hypotheses of the initial hypothesis can be confirmed with the regression equations established.

The regressions of the two measurement points were then compared based on their regression parameters to test whether the influence of the sense of coherence or self-efficacy on mental health or well-being differs between the two measurement points, with the hypothesis that this influence is greater at the first point in time. No differences were observed between the samples at the two measurement points regarding the influence of the two constructs on mental health (*z* = 0.45, *p* = 0.33; *z* = 0.93, *p* = 0.18). Furthermore, no significant difference was observed in the influence of sense of coherence and self-efficacy on well-being between the two measurement points (*z* = 0.30, *p* = 0.38; *z* = −0.65, *p* = 0.26). This renders the second hypothesis unsupported by the data.

The correlation between the sense of coherence and self-efficacy was subsequently examined in the course of further data analysis. A significant Pearson correlation of *r* = 0.58, *p* < 0.001, was identified at the initial measurement point. Additionally, a significant correlation between the sense of coherence and self-efficacy was observed at the second measurement point, with a value of *r* = 0.60, *p* < 0.001. Consequently, Hypothesis 3 can be confirmed based on these findings.

The descriptive results of the questionnaires were then compared at the initial and subsequent measurement time points. Significant differences were identified for the constructs of self-efficacy [*t*(880) = 2.11, *p* < 0.001], mental health [*t*(946) = 4.86, *p* < 0.001] and well-being [*t*(1,077) = −10.13, *p* < 0.001]. The only construct that did not exhibit a statistically significant difference between the two measurement points was sense of coherence, with a *t*-value of 0.45 and a *p*-value of 0.65. A descriptive analysis reveals that the self-efficacy of the second measurement point sample is lower than that of the first. Additionally, mental health outcomes differ, with a greater proportion of students exhibiting depressive symptoms during the pandemic, resulting in poorer mental health compared to the survey conducted after the lifting of social restrictions. Conversely, the second measurement point sample reported higher well-being values. Consequently, hypothesis 4 can be partially confirmed.

## Discussion

The results of this study indicate a positive correlation between the sense of coherence and mental health, thereby confirming part of the initial hypothesis. This lends support to the findings of Langeland et al. (2007) and Torinomi et al. (2022), as well as the observations of Siglen et al. (2007), who identified a negative correlation between the sense of coherence and depressive symptoms. Furthermore, this correlation can be observed not only before and during the pandemic, but also 9 months after the lifting of social restrictions due to the pandemic. Additionally, the positive relationship between self-efficacy and mental health observed in the literature (Bandura et al., 1988) could also be replicated in the analyses presented here, thus confirming another part of the first hypothesis.

The additional hypothesis that the sense of coherence can account for a proportion of the variability in mental health scores was corroborated with an explanatory power of approximately 47% at both measurement points. This finding lends support to the conclusions of Knowlden et al. (2012), who observed that the sense of coherence can account for almost 50% of the variability in mental health scores.

Moreover, the sub-hypothesis that self-efficacy can account for a portion of the variability in mental health scores can be substantiated based on the findings of this study. The proportion of variance explained at the initial measurement time point was 19.7%, while at the second measurement point it was 23.5%. When these values are compared with the explanatory power of the sense of coherence, it is evident that this is higher, with a value of approximately 50% at both measurement times. It can therefore be concluded that self-efficacy can explain a lower proportion of mental health than the sense of coherence.

A correlation of *r* = 0.59 was observed between the sense of coherence and well-being at the initial measurement point, with a correlation of *r* = 0.56 observed at the second measurement point. These findings confirm another aspect of the initial hypothesis and align with previous results reported in the literature (Langeland et al., 2007; Torinomi et al., 2022). With regard to the relationship between self-efficacy and well-being, correlations of *r* = 0.44 and *r* = 0.46 were observed for the initial and subsequent measurement points, thereby confirming a sub-point of the initial hypothesis.

With regard to the linear regression of the sense of coherence on well-being, a notable degree of explanatory power is evident, amounting to approximately 35% at the initial measurement point and approximately 31% at the subsequent measurement point. This serves to confirm another part of the initial hypothesis. The variance explanation of mental health by the sense of coherence was approximately 47% at both measurement times, indicating that the explanatory power of the sense of coherence on well-being is lower. The greater influence of the sense of coherence on mental health than on well-being may be attributed to the fact that a greater number of factors are involved in the assessment of general well-being than in mental health, resulting in a relative decrease in the exploratory power of the sense of coherence.

The final component of the initial hypothesis, which posits that self-efficacy can account for a portion of the observed variability in well-being, can also be corroborated based on the findings of this study. The variance explanation was found to be 19.2% at the initial measurement point and 21.3% at the subsequent measurement point. In previous research, it was observed that the explanatory share of self-efficacy on well-being was 34.9% for men and 29.7% for women (Siddiqui, 2015). These values are higher than those observed in the present study. One potential explanation for this is that, as previously discussed in the context of mental health, individuals had fewer opportunities during the pandemic to engage in self-efficacy-enhancing experiences. Consequently, the variable exhibits a reduced variance, and thus a diminished variance explanation regarding well-being. It is similarly conceivable that external factors determined by the pandemic situation and thus beyond the control of the individuals in question may have exerted an influence on well-being. It is important to note, however, that the differences in the explanatory power at the first and second measurement points are minimal. Consequently, although there is a significant difference, it may have no practical relevance. Furthermore, it is evident that the impact of self-efficacy on well-being is less pronounced than that of the sense of coherence, as the *R*^2^ of the latter exceeds 30% at both measurement points. This can also be attributed to the argument that self-efficacy is highly correlated with the comprehensibility aspect of the sense of coherence.

When considered collectively, these findings substantiate the salutogenesis model proposed by Antonovsky (1979). The protective effect of the sense of coherence on mental health and well-being suggests that sense of coherence may be a significant resource in the context of mental health. Furthermore, self-efficacy may also be a significant factor in the salutogenesis model. In a similar vein, Posadzki and Glass (2009) postulated that self-efficacy can increase an individual’s comprehensibility, thereby facilitating the recognition of the meaning inherent in a given situation and, in turn, enabling more effective coping strategies. Additionally, a notable correlation was identified between the meaningfulness component and self-efficacy, which aligns with the findings of Heslin and Klehe (2006). An alternative perspective on the relationship between sense of coherence and self-efficacy in the context of salutogenesis is that self-efficacy is a key factor in the evaluation of stressors. The evaluation of one’s ability to cope with a stressor as sufficient or insufficient determines the emotional response generated by the stressor, which may range from stress to other negative emotions. A frequent evaluation as insufficient can therefore result in an increased incidence of illness due to the elevated stress levels and thus should be avoided (Antonovsky, 1979). Individuals with a high level of self-efficacy are more likely to perceive their resources as sufficient, as they possess a strong sense of self-belief and have frequently demonstrated the efficacy of their coping strategies. It can therefore be seen that these two concepts are closely related, and that there is value in prioritizing the strengthening of self-efficacy. Firstly, self-efficacy has a direct influence on the salutogenesis model and its associated concepts, such as the sense of coherence. Secondly, there are a number of concrete and relatively straightforward interventions based on self-efficacy that can be readily implemented.

Hypothesis 2, which posits that the sense of coherence or self-efficacy exerts a more pronounced influence on mental health or well-being in the context of a crisis such as the COVID-19 pandemic, was not substantiated by the findings of this study. One potential explanation for this is that both mental health and well-being are influenced by a multitude of factors, with sense of coherence and self-efficacy representing merely two of these influencing variables. Another potential factor is the experience of loneliness (Holm-Hadulla et al., 2021). This is also the reason why greater focus should be placed on research into social interaction opportunities to improve mental health and well-being, especially in the context of crises.

In contrast, hypothesis 3 can be confirmed based on the data, as a positive correlation was found between the sense of coherence and self-efficacy at both measurement times, with correlation coefficients of *r* = 0.58 and *r* = 0.60, respectively. These findings align with existing research, such as that of Posadzki and Glass (2009) and Trap et al. (2016).

The fourth hypothesis, which posits significant differences in values pertaining to coherence, self-efficacy, mental health and well-being between the two measurement points, with values increasing from the first to the second measurement point, can only be partially confirmed. Significant differences were observed between the two time points with regard to self-efficacy, mental health and well-being. However, no significant differences were found in the sense of coherence. It may therefore be posited that the sense of coherence is a more stable construct that was not significantly influenced by the effects of the pandemic. An alternative explanation is that experiencing symptoms of depression is a mechanism for experiencing coherence, for example in the face of excessive demands and chaos, as was the case in the world changed by the COVID-19 pandemic. It is possible that the sense of coherence was not so strongly influenced by the COVID-19 pandemic and its effects, although the new circumstances were experienced as stressful, because the individual sub-facets of the sense of coherence were partially fulfilled.

With regard to the construct of self-efficacy, a notable discrepancy was identified between the two measurement points. However, this was not consistent with the hypothesis, which predicted a reduction in self-efficacy from the first to the second measurement point. Given that the difference in self-efficacy between the two measurement points is only approximately 0.5 points and cannot be logically derived from existing literature, it is possible that the observed difference is only significant due to the size of the sample and that it is not relevant in practice. Mental health exhibited a trend analogous to the fourth hypothesis, with an increase observed between the first and second measurement points. This may be attributed to the fact that students reported experiencing less loneliness and isolation at the second measurement point, which could be considered a contributing factor to the observed decline in students’ general health during the pandemic (Holm-Hadulla et al., 2021; Lim et al., 2022). The opportunity to socialize and interact enabled students to resume their usual lives, which may have contributed to improvements in their mental health and well-being. With regard to well-being, a significant difference in line with the hypothesis was observed in the values of the first and second measurement time points. However, as with mental health, the observed increase from the first to the second measurement point could be attributed to a resumption of the original life with joyful activities. It is therefore vital to further explore the role of social interactions in mental health and well-being.

The findings of Jung et al. (2020) and Torinomi et al. (2022), which indicated that women and younger individuals were more adversely affected by the consequences of the COVID-19 pandemic, are also corroborated by the results of this study. At the initial measurement point, during the pandemic and the resulting social restrictions, significant differences were observed in mental health and well-being between the three demographic variables of age, gender and field of study. Specifically, younger people and women exhibited a higher prevalence of depressive symptoms and a lower level of general well-being compared to their counterparts. At the second measurement point, 9 months after the lifting of social restrictions, no significant differences in mental health and well-being were observed between age groups and genders. However, differences were noted between fields of study. One potential explanation for this is that women and younger individuals were able to rapidly enhance their mental health and, consequently, their well-being by engaging in social interactions. This also underscores the significance of social interactions in the context of mental health and well-being. Consequently, it seems particularly valuable to fortify the mental health of students and younger individuals in general during crises by imparting knowledge.

### Limitations

In this study, self-reporting instruments were employed, which are inherently accompanied by a certain degree of subjectivity. A further limitation is that the two assessments were conducted anonymously. Consequently, data from individuals who participated at both measurement points could not be connected, and only cross-sectional analyses could be conducted. The absence of longitudinal data precludes the possibility of making causal inferences. It should be acknowledged, however, that this procedure may have encouraged a greater number of students to participate, and, moreover, to provide answers that were more candid and transparent. A *t*-test for dependent samples, analogous to that proposed by Holm-Hadulla et al. (2023), was calculated. However, it should be noted that the two measurement times are not the same sample, and that the samples cannot be equated with regard to the demographic variables. It is therefore recommended that the results of these analyses be viewed and interpreted with caution. Furthermore, it is possible that a selection bias may have been introduced, as the email used to recruit respondents already stated that the survey dealt with feelings and experiences in connection with the COVID-19 pandemic. It is therefore possible that individuals who felt particularly burdened by the pandemic and the associated restrictions took part. Conversely, it should be noted that the response rate, at least at the initial measurement point, is commendable for a study of this magnitude that employed email-based recruitment. This conclusion is based on a comparison with a similar study conducted by the German Studierendenwerk (Koob et al., 2021).

### Practical implications

Based on the results of this work, it now appears expedient to derive clear instructions for daily life. It is important for universities to devise a contingency plan to ensure optimal preparedness for unforeseen circumstances (Regehr and Goel, 2020). This plan should integrate the insights gleaned from this study and other research, for instance, guaranteeing that the fundamental tenets of sense of coherence and self-efficacy are upheld when adopting online teaching methodologies. In the aftermath of a crisis, it is crucial to identify the aspects of the situation that were effectively managed and those that were mishandled. This analysis is essential for the development of an enhanced crisis plan for future reference. Additionally, it is vital to maintain an open mind and assess whether strategies employed during the crisis can be applied to the restoration of normalcy. This approach ensures that the lessons learned from the crisis are not lost and can inform future decision-making.

Furthermore, it seems purposeful to place greater emphasis on the aspects that are relevant in the context of increasing and maintaining the sense of coherence and self-efficacy in a variety of settings, including therapy, counseling, schools and universities, as well as in everyday life. In light of the research findings to date and the results of this study, it seems most prudent to disseminate a substantial amount of knowledge about mental health in general and the ways in which it can be influenced. Furthermore, it is recommended that opportunities for positive experiences and social interaction be provided as often as possible, particularly within the student group. It is essential to ensure that these activities are conducted in accordance with the limitations of the current situation. In the event that these conditions are not met, it is of the utmost importance to guarantee that the regulations are clear and readily comprehensible. While the general public cannot be directly involved in significant decision-making processes, it is of the utmost importance to present them in a transparent manner and to elucidate the rationale behind the decision and its relevance in view of the prevailing circumstances. Furthermore, the expansion of online psychotherapy should be encouraged.

## Conclusion

In conclusion, it can be stated that, despite its limitations, the study findings that state that there is a correlation between the constructs sense of coherence, self-efficacy, mental health and well-being can be effectively integrated into the existing literature. It is therefore important to consider ways of strengthening the mental health and well-being of young people, as the data indicated that the constructs sense of coherence, self-efficacy, mental health and well-being had only recovered to a certain extent 6 months after the social restrictions were lifted. This is also a particularly important factor, as a sense of coherence and self-efficacy have been shown to be relevant in the context of influencing mental health and well-being, both in crisis situations and beyond. Therefore, it is recommended that the aforementioned aspects, such as social interactions, which are pertinent to the enhancement and sustenance of coherence and self-efficacy, receive greater consideration in the context of therapeutic, counseling, educational and everyday life settings.

A longitudinal study design is essential to permit the drawing of causal inferences. To illustrate, the time required to recuperate mental health following a crisis could be investigated in greater depth, contingent on the preceding level of the sense of coherence. Furthermore, the stability of the sense of coherence and self-efficacy constructs could be evaluated in such a design.

In conclusion, this study makes a valuable contribution to our understanding of the relationship between sense of coherence or self-efficacy and mental health or well-being in the context of a crisis, particularly among students. This is of particular importance given the evidence that the COVID-19 pandemic has a deleterious and far-reaching impact on the mental health of young people. This study makes a further contribution to the research endeavor into the ways in which this situation might be influenced. It would be beneficial for future research to investigate these relationships in other situations and within different age groups, in order to gain a more generalizable understanding and identify potential differences. Furthermore, a combined analysis of sense of coherence and self-efficacy, and their joint effect on mental health and well-being, would be a valuable addition to the existing literature.

## Data Availability

The raw data supporting the conclusions of this article will be made available by the authors, without undue reservation.
